# Delayed Perforation after Colorectal Cold Snare Polypectomy with Simultaneously Performed Endoscopic Submucosal Dissection: A Case Report and Literature Review

**DOI:** 10.31662/jmaj.2024-0414

**Published:** 2025-06-13

**Authors:** Hironao Matsumoto, Takeshi Yamashina, Masaaki Shimatani

**Affiliations:** 1 Division of Gastroenterology and Hepatology, Kansai Medical University Medical Center, Osaka, Japan

**Keywords:** Chronic kidney disease, Cold snare polypectomy, Corticosteroids, Delayed perforation

## Introduction

Colorectal cancer is the leading cause of cancer-related death among Japanese women and had the highest incidence among all cancers in Japan in 2019 ^(1)^. With advances in endoscopy, numerous adenomatous polyps have been detected in recent years. It is now standard practice to remove such polyps upon detection and arrange for long-term follow-ups to monitor for recurrence, as this is believed to contribute to colorectal cancer prevention ^(2)^.

Cold snare polypectomy (CSP) is a safe means of removal of 5-10 mm sessile polyps without the use of high-frequency current and is now widely implemented because of its ease and safety. A meta-analysis by Shinozaki et al. ^(3)^ showed that CSP requires a significantly shorter procedure time and has a lower delayed bleeding rate than does hot snare polypectomy (HSP). Hence, CSP is recommended as the standard means of resecting small (≤10 mm) adenomatous colorectal polyps ^(4)^. Furthermore, Shinozaki et al. ^(3)^ reported that, in large meta-analyses including a total of 3195 polyps, there were no cases of perforation associated with CSP.

However, four cases of perforation after CSP have been reported more recently ^(5), (6), (7), (8)^.

We report a case of delayed perforation following CSP and concurrent endoscopic submucosal dissection (ESD), requiring surgical intervention.

## Case Report

**Figure 1.**
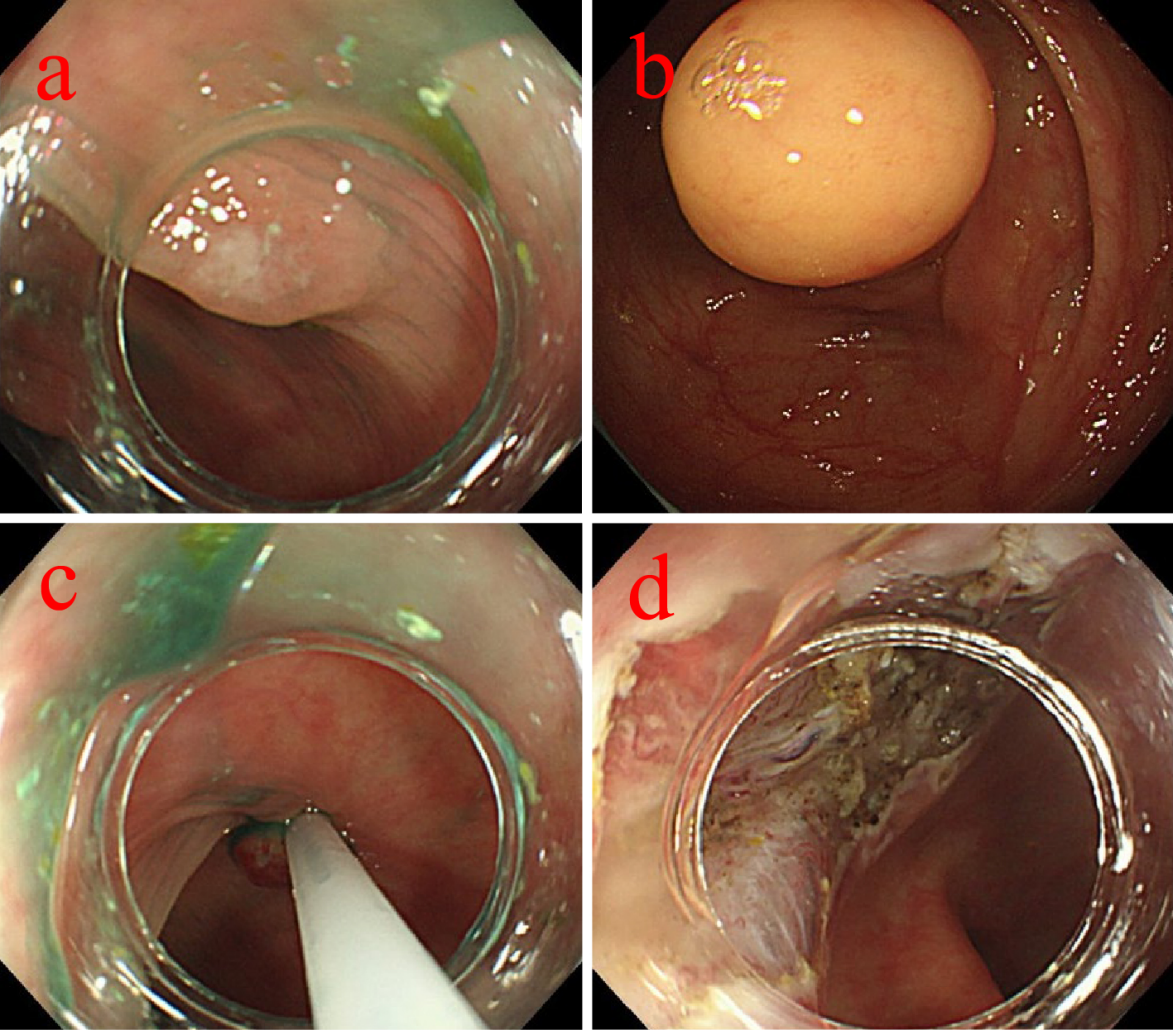
a. The 5 mm Is polyp was found in the transverse colon. b. the 30 mm submucosal tumor was found in the descending colon. c. the 5 mm Is polyp in the transverse colon was resected by CSP. d. ESD was then performed on the 30 mm submucosal tumor in the descending colon.

**Figure 2.**
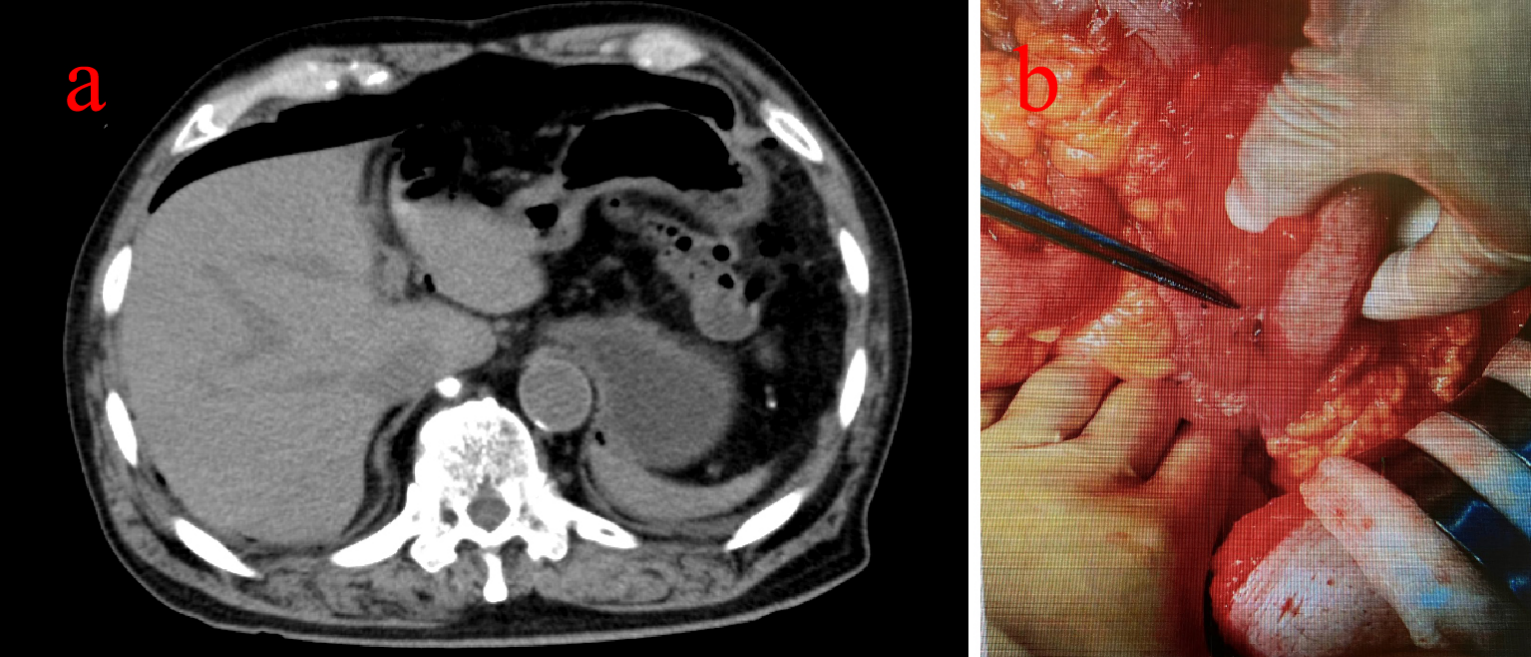
a. Computed tomography showed free air on the liver surface. b. We identified a 5 × 7 mm perforation in the transverse colon.

**Figure 3.**
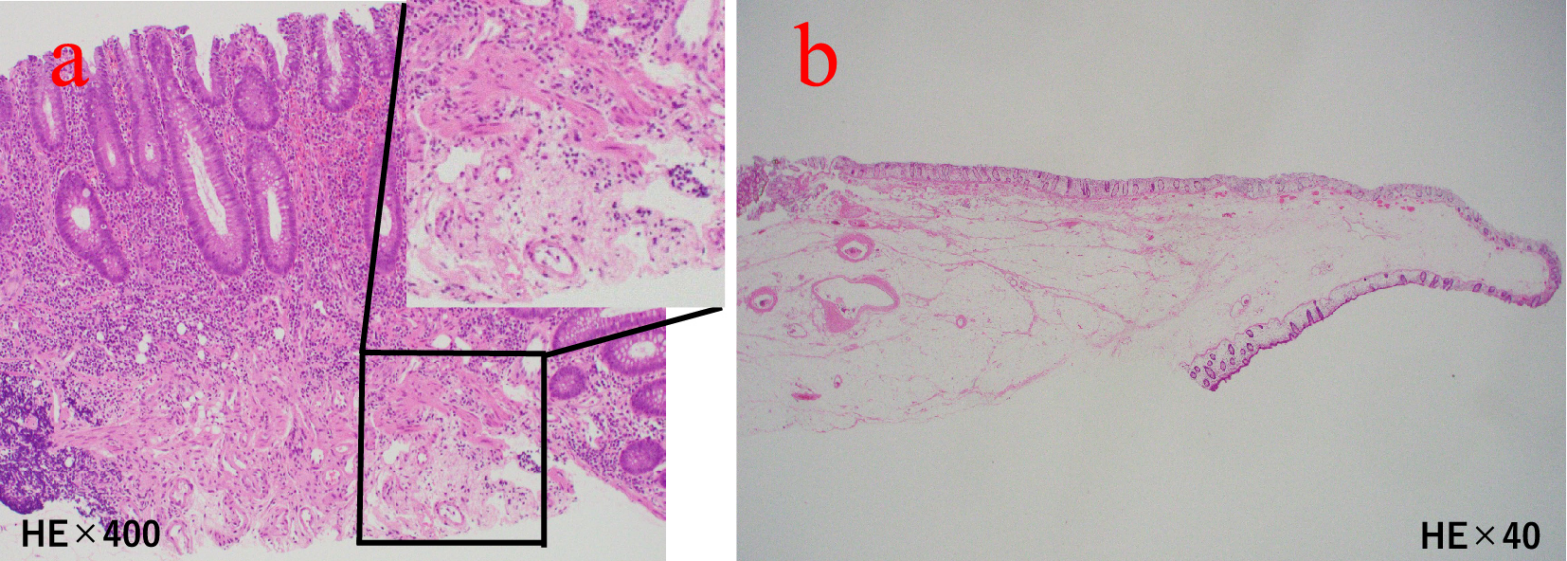
a. Pathological image of a transverse colon polyp is shown. The muscularis propria was not included (hematoxylin and eosin [H＆E] stain, ×40 and ×400). b. Pathological image of a descending colon in the submucosal tumor is shown. The muscularis propria was not included in either case (H＆E stain, ×40).

An 81-year-old man with chronic kidney disease (CKD) stage G4, hypertension, and a history of left nephrectomy for urinary stones underwent colonoscopy for screening purposes for left lower abdominal pain (Table 1). The examination revealed a 30 mm submucosal tumor in the descending colon and a 5 mm polyp in the transverse colon (Figure 1a and b). Although the submucosal tumor was suspected to be a lipoma, we considered the cause of the patient’s left lower abdominal pain. In addition, at the patient’s request, ESD was performed for the tumor, and CSP for the polyp. A 10 mm cold snare was used (Micro-Tech, Nanjing, China). The procedure was challenging due to bowel adhesions from the prior nephrectomy, causing difficulty in stabilizing the colonoscope. The 5 mm polyp was successfully resected by CSP without complications or clipping (Figure 1c). ESD was then performed on the descending colon tumor, completing both procedures in 108 minutes (Figure 1d). As we did not recognize any luminal contents outside the gastrointestinal tract, we judged that there was no obvious intra-operative perforation. The patient initially recovered without symptoms. However, seven hours later, he reported severe abdominal pain with rebound tenderness. Computed tomography revealed free air near the liver, indicating diffuse peritonitis from delayed perforation (Figure 2a). Emergency surgery was performed, revealing yellow, cloudy ascitic fluid and liquid stool in the abdominal cavity. Despite adhesions making exploration difficult, there was no perforation of the sigmoid colon, and a 5 × 7 mm perforation in the transverse colon was located and sutured (Figure 2b). A double-barrel stoma was created, and the abdominal cavity was irrigated. Postoperatively, the patient was treated with fasting, antibiotics, and IV fluids. Recovery was uneventful, and he was discharged 35 days later. Annual endoscopies have shown no recurrence. Pathological examination confirmed the descending colon tumor was a lipoma and the transverse colon polyp an adenoma. No muscularis propria was detected in the specimens (Figure 3a and b).

**Table 1.** Results of Laboratory Investigations on the First Visit.

Complete blood count		Biochemistry		Quantitative Examination	
WBC	3500/μL	TP	6.2g/dL	TP	3
RBC	340×10^4^/μL	Alb	3.7g/dL	Cre	73
Hb	10.3g/dL	Na	1.8mEq/L	TP/Cre	41.1
Ht	33.4%	K	1.5 mEq/L	**Urinalysis**	
PLT	47.8×10^4^/μL	Cl	27 mEq/L	pH	5.5
		AST	19U/L	Protein	(-)
		ALT	12U/L	Glucose	(-)
		IP	3.5U/L	Ketone	(-)
		BUN	37 mg/dL	Occult blood	(-)
		Cre	2.04 mg/dL		
		CCr	22mL/min		
		CRP	0.448mg/dL		

WBC: white blood cells, RBC: red blood cells, Hb: hemoglobin, Ht: hematocrit, PLT: platelets, TP: total protein, Alb: albumin, Na: natrium, K: kalium, Cl: chlorine, AST: aspartate aminotransaminase, ALT: alanine aminotransaminase, IP: inorganic phosphorus, BUN: blood urea nitrogen, Cre: creatinine, CCr: creatinine clearance, CRP: C-reactive protein, pH: potential Hydrogen

## Discussion

We here report a case of delayed perforation 7 hours after an endoscopic procedure during which only CSP and ESD were performed.

De’Angelis et al. ^(9)^ have reported that invasive treatment, such as surgery, is required in patients with a greater than 24-hour delay in the diagnosis of medically induced perforation. Further Panteris et al. ^(10)^ reported that emergency surgery is often recommended when there is a risk of major perforation and signs of peritonitis or abdominal symptoms.

Fortunately, our patient reported abdominal pain 7 hours after the endoscopic procedure, enabling early detection by abdominal examination, blood tests, and computed tomography of his delayed perforation, leading to emergency surgery. Thus, careful observation is necessary after all endoscopic procedures. We responded quickly because we suspected an ESD-related perforation. Such perforations are not rare, whereas there are only a few reports of perforations caused by CSP. Suzuki et al. ^(11)^ assessed the width and depth of CSP and HSP resections and reported that the mucosal defect is smaller after CSP than after HSP. Generally, the depth of resection with CSP is only up to the mucosa, thus it is significantly more superficial than with HSP, making perforation unlikely ^(11)^.

Because perforation after colorectal CSP is not well known, we searched the literature for previous case reports and extracted their characteristics for analysis. A PubMed database search up to March 2024 using the terms (rect*[tiab]) OR (colon*[tiab]) OR (colorect*[tiab]) AND (CSP[tiab]) OR (“cold snare polypectomy”[tiab]) OR (“cold polypectomy”[tiab]) AND (perforation[tiab]) yielded four articles ^(5), (6), (7), (8)^.

We compared and studied five reported cases of perforation associated with CSP, including the present case (Table 2). These included three men and two women with a median age of 62 years. The site of perforation was the transverse colon in three cases and the sigmoid colon in two cases. The polyp morphology was 0-IIa in two cases and Is in three cases, and the polyps’ mean size was 5 mm. The patient’s baseline characteristics were not stated in one case ^(5)^, but two patients had a history of CKD ^(7)^ and two were taking corticosteroids ^(6), (7)^. There were two cases of perforation during endoscopic procedures ^(5), (6)^, both of which were clipped, and three of delayed perforation ^(7), (8)^, one of which was clipped, whereas the other two required emergency surgery ^(7)^, despite which one of them died postoperatively ^(7)^. Perforation during endoscopic procedures is associated with the use of a snare for HSP; pulling the snare sheath with excessive force after initial closure of the snare fails to cut through the tissue may cause perforation ^(3)^.

**Table 2.** Characteristics of Perforation Cases by CSP.

Case (references)	Age (years old)	Gender	Time to perforation	Risk of perforation	Endoscopic resection (perforation) site	Polyp size (mm)	polyp morphology	Treatment for perforation
1 Our case	81	Male	7hours	CKD	Transverse colon	5	Is	Surgery
2 ^(5)^	62	Female	immediately	Unknown details	Sigmoid colon	6	Is	Clipping
3 ^(6)^	61	Female	immediately	corticosteroids	Transverse colon	5	0-Ⅱa	Clipping
4 ^(7)^	77	Male	8days	corticosteroids	Transverse colon	3	Is	Surgery
5 ^(8)^	45	Male	3hours	Nothing paticular	Sigmoid colon	6	0-Ⅱa	Clipping

In the present case, there were adhesions of the bowel, presumably caused by the previous left nephrectomy, which made endoscopic manipulation for ESD difficult and time-consuming. It is possible that over-insufflation and hyperextension of the intestinal tract damaged the post-CSP defect, resulting in micro-perforation. In addition, CKD is reportedly associated with both due to undernutrition and decreased elasticity of the intestinal membrane ^(12)^. Both of these factors may have contributed to our patient’s perforation because he only had one kidney. Indeed, end-stage renal disease is reportedly associated with an approximately two-fold increased risk of perforation during colonoscopic polypectomy ^(13)^. Although our patient had drunk gut lavage fluid before his endoscopy, some stool remained in the intestinal tract. Thus, insufficient bowel preparation was likely one of the factors that intensified his peritonitis.

Oral corticosteroid therapy was not administered in the present case, however, in two cases, the patients were taking corticosteroids due to underlying conditions. A meta-analysis by Narum et al. ^(14)^ reported that the anti-inflammatory and analgesic properties of corticosteroids may mask symptoms and thus possibly delay diagnosis. Corticosteroids also impair tissue repair, thus leading to delayed wound healing, and may increase the OR for gastrointestinal bleeding or perforation by 40% ^(14)^. Therefore, it is important to keep the possibility of delayed perforation after CSP in mind in patients taking corticosteroids. It is even more important to be careful when performing long procedures of ESD or other procedures with defective mucosa, such as after CSP.

The risk of perforation during CSP can be minimized by not forcing the snare if the lesion cannot be resected after strangulation. In addition, as recently reported ^(15)^, in patients on oral steroids and those with renal insufficiency, delayed perforation may be minimized by complete closure of the ulcer with a clip after resection or performing cold EMR with a localized injection.

In conclusion, we experienced a case of delayed perforation seven hours after CSP of a transverse colon polyp in a patient with CKD. A review of published reports suggests that the risk of delayed perforation after CSP may be affected by fragility of the mucosa and poor wound healing in patients with CKD or taking corticosteroids.

## Article Information

### Conflicts of Interest

None

### Author Contributions

Hironao Matsumoto: Conceptualization, writing-original draft. Takeshi Yamashina: Writing-review＆editing.

### Informed Consent

Informed consent was obtained from the patient.

An ethical review was not required for this single-case report.
